# The association between alcohol use and depressive symptoms across socioeconomic status among 40- and 45-year-old Norwegian adults

**DOI:** 10.1186/s12889-015-2479-6

**Published:** 2015-11-19

**Authors:** Priscilla Martinez, Sudan Prasad Neupane, Berit Perlestenbakken, Christina Toutoungi, Jørgen G. Bramness

**Affiliations:** Alcohol Research Group, Public Health Institute, UC Berkeley School of Public Health, Berkeley, CA USA; Norwegian Centre for Addiction Research (SERAF), Institute of Clinical Medicine, University of Oslo, Post box 1039, Blindern, 0315 Oslo Norway; University of Connecticut, School of Medicine, Farmington, CT USA

**Keywords:** Norway, Alcohol use, Heavy drinking, Depressive symptoms, Socio-economic status, Adults, Population-based survey

## Abstract

**Background:**

Little population-based data among middle-aged adults exists examining the relationships between depressive symptoms, alcohol use, and socio-economic status (SES). This study aimed to describe the relationships between depressive symptoms and alcohol use at different levels of SES and to determine differences across SES levels among a population-based sample of 40 and 45 year old adults in Norway.

**Methods:**

This analysis was based on data from two Norwegian health studies conducted in 2000 and 2001, and included community-dwelling Norwegian men and women aged 40 and 45 years. Self-reported frequency and quantity of alcoholic drinks was used to calculate past-year typical quantity of drinks consumed and frequency of 5+ drinks per occasion, or heavy episodic drinking (HED). Depressive symptoms were assessed with the 10-item Hopkins Symptom Checklist, and SES was measured as education level and employment status. To observe the association between depressive symptoms and alcohol use at each level of SES we fitted multinomial logistic regression models using each alcohol outcome as a dependent variable stratified by level of education and employment. To observe differences across levels of SES, we examined the interaction between depressive symptoms and SES level in multinomial logistic regression models for each alcohol measures.

**Results:**

Having depressive symptoms was significantly associated with an increased risk of 5+ typical drinks among people in the lowest (RRR = 1.60, *p* ≤ 0.05) education level, and not among people in the highest. Conversely, significant associations were observed among all levels of employment. For frequency of HED, depressive symptoms was not significantly associated with frequency of HED at any education level. Depressive symptoms was associated with 13+ past year HED episodes among people with no employment (RRR = 1.97, *p* ≤ 0.05), and part-time employment (RRR = 2.33, *p* ≤ 0.01), and no association was observed among people with full-time employment. A significant interaction was observed for depressive symptoms and employment for risk of 13+ past-year HED episodes.

**Conclusions:**

The results show a variety of associations between depressive symptoms and alcohol use among people with lower SES, and suggest type of alcohol use and SES measure may influence the observation of an association between depressive symptoms and alcohol use at different SES levels.

**Electronic supplementary material:**

The online version of this article (doi:10.1186/s12889-015-2479-6) contains supplementary material, which is available to authorized users.

## Background

Depressive symptoms are the most common mood disorder symptoms in the general population [1], and are especially prevalent among people engaging in harmful drinking [2]. While the association between depressive symptoms and alcohol use can vary when different assessments are employed [3], depressive symptoms are consistently associated with the usual quantity of drinks consumed [4, 5] and with heavy episodic drinking [6].

Midlife is an important time to study alcohol use and symptoms of depression because of their impact on the development and course of chronic conditions, many of which have onsets in mid- to- older-age. Heavy alcohol use [7] and depressive symptoms [8] are each well-documented factors for the increased risk of cardiovascular disease. Among middle-aged men, a recent study identified an association between a higher volume of alcohol consumed and a faster cognitive decline [9]. Relatedly, having depressive symptoms or engaging in heavy alcohol use concurrent with a chronic somatic disorder can accelerate disease progression and increase mortality [7, 10]. Identifying circumstances in midlife that have an effect on the relationship between drinking and depressive symptoms could be useful in targeting interventions to groups particularly vulnerable to the negative consequences of concomitant harmful alcohol use and depressive symptoms.

Socio-economic status (SES) may be related to the pattern and severity of alcohol use. In a cross-sectional analysis of data from the GENACIS study, a multi-national study comprising 33 countries of various income levels and over 100,000 participants, higher SES was associated with more frequent alcohol use at both the individual and country level [11]. Similarly, van Oers et al. observed that abstinence from alcohol decreases as education level increases [12] and other studies show that low SES predicts risky alcohol-related behavioural problems and higher alcohol-related mortality [13]. Other studies relate low SES with heavy episodic drinking (HED), often defined as drinking five or more drinks at one occasion, and which is known to confer greater alcohol-related risk than frequent moderate alcohol consumption [14, 15]. This evidence highlights the importance of being distinctive about country and the socio-economic context as well as distinguishing drinking pattern rather than merely any use or frequency of use when studying the relationship between SES and alcohol consumption.

Studies on the relationship between SES and depressive symptoms generally find that low SES is related to a higher prevalence of depressive symptoms [16]. Several indicators of SES, including one’s own and parent’s education, employment, income and disability benefits are associated with antidepressant use. Von Soest et al. [17] showed that antidepressant use as a proxy measure for depression was associated with low levels of income and education among Norwegian adults. In contrast, the Epidemiologic Catchment Area Study in the US found few differences in the rate of a major depressive episode across socio-economic status, observing a statistically insignificant higher incidence of MD among the unemployed compared to those with employment [18]. However, fulfilling criteria for MD is more stringent than experiencing symptoms of depression, such that the relationship between depressive symptoms and SES may be different than that of MD and SES. Similarly, a variety of metrics have been employed to measure SES, and different measures can produce different results. A recent study from Sweden by Linander et al. identified longitudinal associations of the SES measures of financial strain and living on social welfare with psychological distress, and no associations between either occupation or education and psychological distress [19]. Another Swedish study showed that depression is more strongly related to alcohol related problems in middle-aged and older individuals compared to younger adults [20]. In a high income, egalitarian country such as Norway, the association between SES and health has often been measured by educational level and employment status [17, 21]. However, studies such as Linander’s suggest employing a variety of measures may improve the validity of the SES construct, even in a high income, low inequality setting.

Although there is a substantial body of work on the associations between alcohol use and depressive symptoms [2], and each of these constructs and SES, there is less research on the relationship of all three, and in particular potential differences in the associations between depressive symptoms and alcohol use at different levels of of SES. Thus, this study aimed to observe the association between depressive symptoms and alcohol use at each level of SES, and to observe differences across levels of SES in the association between depressive symptoms and alcohol use in a population-based sample of 40- and 45- year-old community-dwelling Norwegians.

## Methods

### Sample

We used data from two Norwegian health studies conducted in 2000 and 2001: The Health Study in Oppland and Hedmark (OPPHED), and the Oslo Health Study (HUBRO). Næss and colleagues have described in detail the study design and procedures for these surveys [22]. In short, The OPPHED Study invited 25,000 people to participate and enrolled 6142 from Oppland and 6362 from Hedmark for a total response rate of 50 %. The HUBRO Study invited 40,000 people to participate and enrolled 18,770 (46 %) participants. Both surveys sampled participants from the following birth cohorts: 1925, 1940, 1955, 1960, and 1970. This study used data from the 1955 and 1960 birth cohorts in both studies. Participants at the time of the survey were 40 and 45 years old, respectively. Between the two surveys, a total of 24,997 individuals born in 1955 and 1960 were eligible to participate, and 12,261 were enrolled, garnering a total response rate of 50.5 %.

The research complied with the Helsinki Declaration. Both OPPHED and HUBRO studies were approved by the Norwegian Data Inspectorate and the Regional Committees for Medical Research Ethics in Norway. All participants signed an informed consent form prior to data collection.

We included in this study a total of 10,872 participants who took a medical examination and had valid responses for all of the following items in the survey questionnaire: years of education, employment status, the Hopkins Symptom Checklist-10 item version (HSCL-10), frequency of alcohol use, past year typical quantity consumed and past year frequency of five or more drinks. We excluded individuals with incomplete information on the following items: HSCL-10 (*n* = 752), lifetime alcohol-use and use during the past year (*n* = 130), employment (*n* = 187), and education (*n* = 198). For the 532 subjects with 1 or 2 missing items on the HSCL-10, we imputed the sample mean for missing item values. A description of missing data and significant differences on a variety of characteristics between persons with and without missing data is provided as Additional file 1.

### Measures

#### Depressive symptoms

We used the validated Norwegian version of the 10-item Hopkins Symptom Checklist (HSCL) to determine the occurrence of depressive symptoms (21). To each of the ten items participants identified the level of symptom experienced over the past week on a scale from 1 (not at all) to 4 (extremely). We calculated sum scores and averaged over the number of items, producing a range of scores from 1 (lowest) to 4 (highest). Consistent with standard cut-offs, we considered a score equal to or greater than 1.85 indicative of depression [23].

#### Alcohol measures

We classified participants as current drinkers if they reported consuming any alcohol in the last 12 months; those who were abstinent during the previous 12 months we classified as either lifetime abstainers if they reported no lifetime alcohol use or former drinkers if they reported drinking alcohol in their lifetime but no use in the last 12 months. Alcohol consumption among drinkers was measured for past year use.

Typical quantity of drinks per drinking occasion over the previous year was assessed with the question “How many drinks do you typically have when you are drinking?”, and responses were recorded as the number of drinks. For the analyses, typical number of drinks per occasion was converted into a categorical variable with the following categories: 1–2 drinks, 3–4 drinks, 5+ drinks.

Frequency of five or more drinks per occasion, or heavy episodic drinking, was assessed with the question “How often over the last year did you have five or more drinks in a day?”, and responses were recorded as the number of times. For the analyses, frequency of HED was used as a categorical variable with the following categories: none, 1–6 times per year, 7–12 times per year, 13 + times per year. Frequency of HED was categorized as such to reflect less than monthly, monthly, or greater than monthly frequency of HED in the past year. Monthly frequency of HED has been commonly used in previous studies [24].

#### Socioeconomic status

We measured socioeconomic status as education level and employment status. Education was measured as years of education completed. We categorized years of education into tertiles. The first tertile included 0–11 years of education, the second included 12–15 years and the third included 16 or older years. According to the educational system present in Norway for the 1955 and 1960 birth cohorts, the first tertile corresponds with education up to the completion of secondary education in Norway; the second tertile corresponds with completion of bachelor-degree level education; and the third tertile indicates a higher university level education. Employment status was assessed with the question “Are you currently in paid employment?” with possible responses of full time, part time and not currently in paid employment.

#### Other measures

Self-reported health status was assessed on a four point scale ranging from “bad” to “very good”. Current, previous and ever smoking tobacco was also queried. Additional covariates included area of residence (Oslo, Hedmark or Oppland), and cohabitation with a spouse or partner.

#### Statistical analysis

Descriptive statistics are presented as frequencies and percentages. Differences in alcohol use and depressive symptoms across education and employment were tested with chi-square tests of independence. To observe the association between depressive symptoms and alcohol use within each level of education and employment, we stratified by education and employment and fitted multinomial regression models for typical quantity of drinks consumed and frequency of past year HED separately. Adjusted models included age, gender, cohabitation status, area of residence, self-reported health status and smoking. To observe whether the association between depressive symptoms and alcohol use measures varied by education and employment status, we fitted adjusted multinomial logistic regression models for each alcohol outcome for education and employment separately, including an interaction term between depressive symptoms and the SES measure. Relative Risk Ratios and 95 % Confidence Intervals are presented, and analysis was carried out using STATA version 11.

## Results

The sample was comprised of 5954 (54.8 %) women and 5509 (50.1 %) people aged 40. Half the sample (51.2 %) resided in Oslo, with the remaining half evenly split between Hedmark (24.8 %) and Oppland (24.1 %). Over three-quarters (75.9 %) of the sample were cohabitating with a spouse or partner. Under a fifth of the sample (17.4 %) reported “not good” health, while 58.4 and 22.8 % reported good and very good health, respectively. Approximately a third of the respondents (32.9 %) were current smokers, and a quarter of the respondents (26.1 %) were previous smokers. Overall, 10.8 % of the sample reported levels of depressive symptoms above the standard cut-off. Approximately 3.5 % were lifetime abstainers and the same proportion was formers drinkers; the remaining 92.9 % were current drinkers. Self-reported drinking is reported in Table 1.

**Table 1:** Depressive symptoms and alcohol use by education and employment

		Education			Employment		
	Total	Lowest tertile	Middle tertile	Highest tertile	None	Part-time	Full-time
	n (%)	n (%)	n (%)	n (%)	n (%)	n (%)	n (%)
Depressed	1178 (10.8)	465 (15.1)	444 (10.4)	269 (7.7)**	387 (35.0)	222 (11.2)	569 (7.3)**
Lifetime abstainer	388 (3.6)	172 (5.6)	139 (3.3)	77 (2.2)**	143 (12.9)	69 (3.5)	176 (2.3)**
Former drinker	383 (3.5)	144 (4.7)	136 (3.2)	103 (2.9)**	98 (8.9)	75 (3.8)	210 (2.7)**
Current drinker	10,101 (92.9)	2763 (89.7)	4000 (93.6)	3338 (94.9)**	866 (78.2)	1844 (92.8)	7391 (95.0)**
Typical drink quantity^a^							
1–2 drinks	5665 (56.1)	1354 (49.0)	2172 (54.3)	2139 (64.1)**	460 (53.1)	1277 (69.3)	3928 (53.2)**
3–4 drinks	3252 (32.2)	912 (33.0)	1344 (33.6)	996 (29.8)*	256 (29.6)	475 (25.8)	2521 (34.1)**
5+ drinks	1184 (11.7)	497 (18.0)	484 (12.1)	203 (6.1)**	150 (17.3)	92 (5.0)	942 (12.8)**
HED frequency^a^							
None	3356 (33.2)	987 (35.7)	1275 (31.9)	1094 (32.8)*	371 (42.8)	929 (50.4)	2056 (27.8)**
1–6 times	3949 (39.1)	1093 (39.6)	1573 (39.3)	1283 (38.4)	269 (31.1)	657 (35.6)	3023 (40.9)**
7–12 times	1418 (14.0)	342 (12.4)	597 (14.9)	479 (14.4)*	106 (12.2)	151 (8.2)	1161 (15.7)**
13+ times	1378 (13.6)	341 (12.3)	555 (13.9)	482 (14.4)*	120 (13.9)	107 (5.8)	1151 (15.6)**

Note: *P*-values are from chi-square tests of independence

^a^Frequencies and percentages out of current drinkers (past year use) *p ≤ 0.05, **p ≤ 0.001

### The distribution of depressive symptoms and alcohol use across education and employment

The distribution of depressive symptoms and alcohol use across the different levels of education and employment are presented in Table 1. People in the lowest tertile of years of education and with no current employment had the highest rates of depressive symptoms at 15.1 and 35.0 %, respectively (*p* < 0.001). Current drinkers were most common among people in the highest tertile of education (94.9 %) and with full-time employment (92.9 %). The number of typical quantity of drinks was negatively associated with level of education, where having 1–2 drinks was most common among people in the highest education tertile (64.1 %) vs. people in the lowest tertile (49.0 %, *p* < 0.001) and having a typical quantity of drinks of 5+ was highest among people in the lowest tertile of education compared to people in the highest, at 18.0 % vs. 6.1 %, respectively, (*p* < 0.001). Conversely, there was a positive association between frequency of past year HED with education, where people in the highest education tertile had a higher proportion of having 7–12 past year episodes HED than people in the lowest (14.4 % vs. 12.4 %, *p* < 0.05). There were no differences across education for having 1–6 HED episodes in the past year. We noted a similar observation for HED frequency across levels of employment.

### Associations between depressive symptoms and alcohol use within education and employment levels

Unadjusted and adjusted associations between depressive symptoms and alcohol use across education and employment are presented in Tables 2 and 3. For typical number of drinks within levels of education, adjusted models showed, having depressive symptoms was significantly associated with an increased risk of having consumed five or more typical drinks relative to having 1–2 typical drinks among people in the lowest (RRR = 1.60, *p* ≤ 0.05) and middle (RRR = 1.68, *p* ≤ 0.05) tertiles of education, whereas no significant association was observed among people in the highest tertile of education. Conversely, significant associations between depressive symptoms and having five or more typical drinks relative to 1–2 typical drinks were observed among both people with no current employment (RRR = 2.00, *p* < 0.01), part-time employment (RRR = 2.06, *p* ≤ 0.05) and people with full-time employment (RRR = 1.35, *p* < 0.05). For frequency of HED, adjusted models showed depressive symptoms was not significantly associated with any level of frequency of HED within any of the levels of education. Within employment levels, depressive symptoms was associated with having 13+ past year HED episodes relative to 1–6 HED episodes among people with no employment (RRR = 1.97, *p* < 0.05), and part-time employment (RRR = 2.33, *p* ≤ 0.01) in adjusted models, whereas no association was observed among people with full-time employment.

**Table 2:** Unadjusted and adjusted relative risk ratios for the association between past year typical drink quantity^a^ and depressive symptoms by education and employment among middle-aged community dwelling Norwegians

Depressed group	3–4 drinks typical		5+ drinks typical	
	Unadjusted	Adjusted	Unadjusted	Adjusted
	RRR (95 % CI)	RRR (95 % CI)	RRR (95 % CI)	RRR (95 % CI)
*Education*				
Lowest tertile	1.00 (0.78,1.28)	1.10 (0.83,1.46)	1.50 (1.14,1.98)*	1.60 (1.13,2.34)*
Middle tertile	1.29 (1.02,1.63)*	1.18 (0.91,1.53)	2.09 (1.56,2.79)**	1.68 (1.18,2.39)*
Highest tertile	1.07 (0.80,1.44)	1.03 (0.75,1.42)	2.23 (1.45,3.45)***	1.58 (0.94,2.67)
*Employment*				
None	1.42 (1.03,1.97)*	1.21 (0.82,1.79)	2.75 (1.88,4.02)***	2.00 (1.22,3.26)**
Part-time	1.26 (0.90,1.77)	1.09 (0.75,1.60)	4.52 (2.80,7.30)***	2.06 (1.12,3.79)*
Full-time	1.15 (0.95,1.40)	1.11 (0.90,1.37)	1.53 (1.19,1.97)**	1.35 (1.01,1.80)*

Note: Adjusted models included age, gender, cohabitation status, area of residence, self-reported health status and smoking

^a^Reference group = 1–2 drinks typical *p ≤ 0.05 **p ≤ 0.01 ***p < 0.001

**Table 3:** Unadjusted and adjusted relative risk ratios for the association between past year HED frequency^a^ and depressive symptoms by education and employment among middle-aged community dwelling Norwegians

Depressed group	No HED past year		7–12 past year HED episodes		13+ past year HED episodes	
	Unadjusted	Adjusted	Unadjusted	Adjusted	Unadjusted	Adjusted
	RRR (95 % CI)	RRR (95 % CI)	RRR (95 % CI)	RRR (95 % CI)	RRR (95 % CI)	RRR (95 % CI)
*Education tertile*						
Lowest	1.20 (0.94,1.54)	1.10 (0.82,1.47)	1.16 (0.82,1.64)	1.34 (0.90,1.98)	1.27 (0.90,1.79)	1.39 (0.94,2.07)
Middle	1.12 (0.87,1.45)	1.08 (0.82,1.44)	0.96 (0.69,1.34)	1.00 (0.70,1.44)	1.48 (1.09,2.00)*	1.25 (0.88,1.79)
Highest	0.94 (0.69,1.29)	0.90 (0.64,1.27)	0.89 (0.58,1.35)	1.00 (0.64,1.56)	1.25 (0.86,1.83)	1.27 (0.83,1.94)
*Employment*						
None	1.20 (0.86,1.68)	0.92 (0.61,1.38)	1.14 (0.72,1.81)	0.99 (0.57,1.73)	2.39 (1.57,3.64)***	1.97 (1.12,3.46)*
Part-time	1.23 (0.88,1.72)	1.13 (0.78,1.63)	1.56 (0.92,2.62)	1.56 (0.86,2.82)	3.44 (2.11,5.62)***	2.33 (1.27,4.29)**
Full-time	1.09 (0.88,1.35)	1.03 (0.82,1.30)	0.95 (0.73,1.24)	1.05 (0.79,1.40)	0.99 (0.75,1.29)	1.01 (0.76,1.36)

Note: Referent group of 1–6 HED past year episodes chosen by STATA “mlogit” command default setting to choose the most frequent outcome as the base outcome. Adjusted models included age, gender, cohabitation status, area of residence, self-reported health status and smoking

^a^Reference group = 1–6 HED past year episodes *p < 0.05 **p < 0.01 ***p < 0.001

### Differences in associations between depressive symptoms and alcohol use across education and employment

Assessing the interaction between depressive symptoms and education and employment separately to assess differences in the associations between depressive symptoms and measures of alcohol use at different levels of SES revealed only one significant difference. We observed that depressive symptoms affect the relative risk of having 13+ past-year HED episodes vs. 1–6 episodes differently across employment strata. In the adjusted model, the relative risk of depressive symptoms on 13+ past year HED episodes vs. 1–6 episodes among people with part-time employment was greater than the relative risk of depressive symptoms on 13+ past year HED episodes vs. 1–6 episodes among people with full-time employment (RRR = 1.95, *p* = 0.03). Among people with no employment, the relative risk of depressive symptoms on 13+ past year HED episodes vs. 1–6 episodes was greater than the relative risk of depressive symptoms on 13+ past year HED episodes vs 1–6 episodes among people full-time employment (RRR = 1.76, *p* = 0.04).

## Discussion

This study observed associations between depressive symptoms and two measures of alcohol use at different levels of education and employment as measures of SES. We observed associations between depressive symptoms and typical quantity of drinks consumed in the past year at the lowest and middle levels of education, and associations at all levels of employment. We observed no associations between depressive symptoms and past-year HED frequency at any level of education, and associations among people with no and part-time employment only. We also found evidence showing differences in the associations between depressive symptoms and frequency of past-year HED across levels of employment, where people with no and part-time employment had stronger associations than people with full-time employment. The results show mixed support for the hypothesis that there is a stronger association between depressive symptoms and alcohol use among people with lower SES, and suggest type of alcohol use and SES measure may influence the observation of an association between depressive symptoms and alcohol use at different SES levels.

Our findings that some associations between measures of alcohol use and depressive symptoms were statistically significant within particular indicators of lower SES is consistent with epidemiologic work showing that people experiencing greater social disadvantage suffer poor health outcomes, including somatic [25] and mental health disorders. Suggested mechanisms driving this disparity are a differential in exposure to chronic stressors [26, 27] or enhanced vulnerability to negative health- related consequences when exposed to risk factors [28]. Either of these mechanisms is plausible regarding the relationship between alcohol use and depression, and warrant study in future research to elucidate mechanisms driving the socioeconomic disparity in the association between alcohol use and depressive symptoms.

The association between alcohol use measured as typical quantity of drinks and depressive symptoms is consistent with previous studies. Graham et al. reported a small, positive relationship between volume of drinking and depression in a population-based survey of over 14,000 Canadians [3]. In a sample of adults aged 65 or older, Graham and colleagues similarly reported an association between alcohol volume and psychological well-being [4]. However, consistent with our results of a stronger relationship between depressive symptoms and HED compared to typical quantity of drinks, Graham’s study further reported the strongest relationship between heavy drinking and poorer psychological well-being [4].

For typical number of drinks consumed, an association with depression was observed at lower levels of education and not at higher levels, where depression was associated with an increased likelihood of consuming a higher number of typical drinks. We did not observe this pattern for employment, that is, we observed an association between depression and typical number consumed at all levels of employment. These contradictory findings likely reflect that no single measure of socioeconomic status can provide a complete picture of socioeconomic position. A recent study from Australia estimated the relative magnitude of socioeconomic inequalities in health by income, education and an area-based socioeconomic index, and found substantial variations according to type of SES measure [29]. Our different findings between education and employment could potentially also reflect the differential effects of proximal versus distal socioeconomic conditions on the relationship between depressive symptoms and the typical number of drinks consumed; namely the impact of having current, disposable income and gainful employment versus the impact of a history or the long-term condition of low SES as represented by lower educational attainment. The latter might suggest sustained, chronic low SES, as opposed to the transient state of being in a temporary financially disadvantaged position by being without current employment. This temporality may be an important aspect of the impact of SES on the relationship between depression and alcohol use, especially when alcohol consumption is measured as typical quantity of use, and should be considered in future studies of the effect of SES on the relationship between alcohol use and depressive symptoms.

In contrast to the observations we identified for the association between depressive symptoms and typical quantity consumed by education and employment, the associations between depressive symptoms and HED were only observed for the employment measure of SES, where depression was associated with more frequent HED episodes among people with no and part-time employment. Moreover, we observed an interaction between employment and depressive symptoms on the effect on risk of past-year HED frequency, where stronger associations between depressive symptoms and having 13+ past-year HED episodes were observed among people having no and part-time employment compared to those in full-time employment. This suggests employment may moderate the association between depressive symptoms and HED frequency, where people with lower level so employment are at greater risk of HED when depressive symptoms are present than people at higher levels of employment. This may speak to the measure of HED as a more acutely harmful pattern of drinking for health than any use or typical consumption [30], and is supported by the consistent evidence linking HED with depressive symptoms across different measures of depression and depressive affect [4, 6]. This supports the development of prevention interventions for people not engaged in gainful employment or in part-time employment who are experiencing depressive symptoms in an effort to prevent or lessen the frequency of HED episodes and their potential negative consequences.

The limitations of this study deserve mention. First, the response rate was on the lower end of what is considered acceptable for population-based surveys, and there were significant differences across various sociodemographic characteristics between those who were missing data on key variables and excluded from the dataset and those who had valid responses. This low response rate and the systematic differences in missing data mean this sample is not representative of the Oslo, Hedmark and Oppland municipalities from which they were drawn. Relatedly, the survey was conducted nearly 15 years ago when recorded adult per capita pure alcohol consumption was 5.7 L compared to 6.1 L in 2014, but drinking patterns and drinking norms have changed only marginally among middle-aged drinkers in Norway (www.ssb.no). Thus, generalizations to the current, middle-aged general populations of these regions should be done with some care. Also, there is a selection bias towards those who are more likely to participate in health surveys and respond to questions on depressive symptoms and alcohol. But in terms of the associations we investigated, beyond the low response rate, the pattern of missing data may introduce bias into the observed associations. That people who were unemployed and less well educated were often missing responses on depressive symptoms and alcohol use measures indicates we might have underestimated the effect sizes of SES. This would occur if levels of depressive symptoms and alcohol use were higher among the unemployed and less well educated, as we observed in the sample analyzed. In such a case, the exclusion of these cases may have biased the associations towards the null. However, since no data exists for non-responders to examine this possibility, we also acknowledge that no bias or a bias against the null is possible. Self-reports of alcohol use are always subject to under-reporting, and may therefore below estimates of true alcohol consumption. Moreover, the frequency of past-year HED was assessed as the number of times overall and not according to a monthly or weekly timeframe. While the categories we constructed for the frequency of past-year HED were an attempt to correspond these numbers to monthly or less than monthly HED frequencies, they are not precise and limit this measures utility. In addition to more refined measures of HED frequency, using other measures of alcohol consumption in future studies, particularly standardized measures and gender-specific measures, would help further our understanding of the relationships between depressive symptoms, alcohol use, and socio-economic status. Our measures of socio-economic status as employment status and years of education are crude, and additional measures such as household and individual income, and parent’s background would serve to refine this measure. Finally, as this is cross-sectional data we cannot infer about the causality between depressive symptoms and alcohol use, or the point on this causal pathway at which the effect of SES takes place.

## Conclusions

Overall, these findings suggest people with lower levels of education and without full-time employment to have a greater risk of experiencing depressive symptoms when consuming higher amounts of alcohol, either as their typical mode of intake or as heavy episodic drinking. Alternatively, it suggests that people experiencing depressive symptoms may be at a greater risk of developing harmful drinking patterns. Also, in either case, identifying that groups with lower SES are more vulnerable to alcohol-related and mental health harms supports calls for prevention interventions targeting this group, especially because this group may be less likely to seek and receive treatment for their maladies [17]. Our findings also suggest that having several and varied measures of SES and alcohol use are an important methodological consideration when studying the impact of SES on the relationship between depressive symptoms and alcohol use.

## Additional file

Additional file 1:
**Description of missing data.** (PDF 82 kb)

## Abbreviations

HED: heavy episodic drinking
HSCL-10: Hopkins Symptoms Checklist-10
HUBRO: The Oslo Health Study
MD: major depression
OPPHED: The Health Study in Oppland and Hedmark of Norway
RRR: relative risk ratio
SES: socioeconomic status
