# Supplementary material for: The association between alcohol use and depressive symptoms across socioeconomic status among 40- and 45-year-old Norwegian adults
Source: BMC Public Health. 2015 Nov 19;15:1146. doi: 10.1186/s12889-015-2479-6 (PMC4653901; doi:10.1186/s12889-015-2479-6)
Supplement: Additional file 1: — Description of missing data. (PDF 82 kb) [file 12889_2015_2479_MOESM1_ESM.pdf]

SUPPLIMENTARY MATERIAL

Of the original 12,621 individuals, 752 (6%) were missing 3 or more items on the HSCL-10. These 752 individuals were therefore excluded from the dataset used for analysis. They did not differ significantly from those who had 2 or less missing HSCL-10 items by gender, age or smoking status. They were, however, more likely to not be cohabitating with a partner (7.1% vs.4.5%, p<0.000), to self-report “bad” health compared to very good health (17.8% vs. 2.6%,respectively, p<0.000), to be an Oslo resident vs. Hedmark or Oppland (6.8% vs. 4.6% vs. 5.6%, respectively, p<0000), to be unemployed vs. having part-time or full-time employment (10.7% vs. 6.1% vs. 4.3%, p<0000), and have on average 1.7 fewer years of education (12.0 vs. 13.7 years, p<0.000).. One-hundred and thirty subjects (1.0%), were missing data on frequency of alcohol use, including lifetime and past year use, and were excluded. Among subjects who reported any alcohol use, 616 (5.3%) were missing values for typical quantity consumed over the past year, and 936 (8.0%) were missing data on past year frequency of five or more drinks. People missing responses for past year typical quantity consumed did not differ from thosewith responses by age or gender. They were, however, more likely to not be cohabitating with apartner (8.4% vs. 4.3%, p<0.000), to have currently smoked vs. previously vs. never smoke (5.8% vs. 4.1% vs. 5.1%, p=0.007, respectively), to be an Oslo resident vs. Hedmark or Oppland (6.1% vs. 4.2% vs. 4.6%, p<0.000, respectively), to self-report “bad” health compared to very good health (18.0% vs. 3.5%, respectively, p<0.000), to be unemployed vs. having part-time or full-time employment (10.3% vs. 4.1% vs. 4.1%, respectively, p<0.000), and had on average 2.0 fewer years of education (11.8 vs. 13.8 years, p<0.000). The 936 people with missing values for past year frequency of drinking five or more drinks did not differ by age. However, they were more likely to be women (8.5% vs. 7.4%, p=0.03), to not be cohabitating (11.7% vs. 6.8%, p<0.000), to be current smokers vs. previous vs. never smokers (9.2% vs. 6.6% vs. 7.4%, p<0.000), to self-report “bad” health compared to

very good health (23.0% vs. 5.7%, $p<0.000$), to be an Oslo resident vs. Hedmark or Oppland (10.1% vs. 5.4% vs. 6.5%, respectively, $p<0.000$), to be unemployed vs. have part-time or full-time employment (14.7% vs. 7.7% vs. 6.4%, $p<0.000$), and had on average 1.6 fewer years of education (12.2 vs. 13.8, $p<0.000$). Finally, 187 (1.5%) and 198 (1.6%) were missing data for employment and education, respectively, and were also excluded.
